# MED6/357: Looking over the Horizon: An Internet-based International Course in Comparative Healthcare Management

**DOI:** 10.2196/jmir.1.suppl1.e49

**Published:** 1999-09-19

**Authors:** U Lerch, M von der Leyen, L Dierks, T Rathwell, P Berman, D Burnett, S MacDonald, M Mantyranta, M Moffatt, T O'Sullivan

**Affiliations:** ^1^Hannover Medical School, HannoverGermany

**Keywords:** Education, Distance, International Educational Exchange, Education, Medical, Graduate, Internet

## Abstract

**Introduction:**

In 1998, the unique and experimental "Looking over the horizon - An Internet-based International Course in Comparative Healthcare Management" started. The course is a component of the larger project on "Promoting International Co-operation and Understanding in Healthcare Management". It is funded by the Canada-European Community Program for Co-operation in Higher Education and Training - a joint initiative between the Canadian Government and the European Commission.

**Methods:**

The purpose of the course is to enable graduate students from participating countries - Canada, Germany, Finland, and Ireland - to become better healthcare managers by learning more about their own and each others' healthcare systems and management processes. The course is structured around an introductory module (Healthcare Systems) and four theme modules: Financing and Funding, Healthcare Delivery Issues, Impact of Health System Reform, and Evidence-based Management. The technology used for the delivery of the course is WebCT, a web-based distance learning software developed at the University of British Colombia. WebCT provides a large number of functions both for students - e.g.,

e-mail, bulletin boards, chat rooms, and calendar - and for instructors - e.g., student tracking, page tracking, chat room log files, and marking management. Instructors are able to design the whole course, receive students' assignments, and post their assessments, via the World Wide Web.

**Results:**

From January to April 1999, 25 students participated in the second course (19 students in the first course in 1998). The tracking function of the WebCT system was used to get some data about the students' activities. In 15 weeks, the students read an average number of 585 (min. 137, max. 806) contributions -- i.e. the sometimes very profound messages posted by students and instructors --, whereas they posted 26 (min. 6, max. 67) own contributions. The high activity of students is a typical characteristic of this student-centred course. Students have the opportunity and responsibility to be both students and teachers for the others. The role of the instructor, however, changes from that of the source of knowledge to that of a supervisor. Therefore students themselves can significantly increase the quality of the course.

**Discussion:**

So far, 44 students participated in the course. For all of them, it has been a valuable experience of learning, which both increased their knowledge of national and international management issues and improved their technical skills on the field of the new medium Internet. The possibility to provide international courses via the Internet gives a new dimension to world-wide medical education and should be used very intensively. For the part of "Looking over the Horizon", the course is planned to expand on more students and even more countries.

